# Retraction: Dietary patterns in adults following the Christian Orthodox fasting regime in Greece

**DOI:** 10.3389/fnut.2024.1520555

**Published:** 2024-11-05

**Authors:** Frontiers Editorial Office

The publisher retracts the March 7 2022 article cited above.

Following publication, concerns were raised regarding the provenance of some data used in this study. Specifically, during the investigation, which was conducted in accordance with Frontiers' policies, the authors informed us that they lacked appropriate authorization for use of the relevant data. Unauthorized data use is a breach of Frontiers' guidelines and those of the Committee on Publication Ethics. As such, this article is being retracted.

This retraction was approved by the Chief Executive Editor of Frontiers. The authors agree to this retraction.

